# *TAP1* I333V gene polymorphism and type 1 diabetes mellitus: a meta-analysis of 2248 cases

**DOI:** 10.1111/jcmm.12244

**Published:** 2014-03-21

**Authors:** Yan-Yan Li, Wei Gao, Si-Si Pang, Xiao-Yan Min, Zhi-Jian Yang, Hui Wang, Xin-Zheng Lu, Lian-Sheng Wang, Xiang-Ming Wang, Yun Qian, Chuan-Wei Zhou, Jun Wu, Ai-Ling Chen

**Affiliations:** a Department of Geriatrics, First Affiliated Hospital of Nanjing Medical UniversityNanjing, China; b Department of Cardiology, First Affiliated Hospital of Nanjing Medical UniversityNanjing, China

**Keywords:** Transporter associated with antigen processing 1, I333V, polymorphism, type 1 diabetes mellitus

## Abstract

*Transporter associated with antigen processing 1* (*TAP1*) I333V gene polymorphism has been suggested to be associated with type 1 diabetes mellitus (T1DM) susceptibility. However, the results from individual studies are inconsistent. To explore the association of *TAP1* I333V gene polymorphisms with T1DM, a meta-analysis involving 2246 cases from 13 individual studies was conducted. The pooled odd ratios (ORs) and their corresponding 95% confidence intervals (95% CIs) were evaluated by a fixed-effect model. A significant relationship was observed between *TAP1* I333V gene polymorphism and T1DM in allelic (OR: 1.35, 95% CI: 1.08–1.68, *P* = 0.007), dominant (OR: 1.462, 95% CI: 1.094–1.955, *P* = 0.010), homozygous (OR: 1.725, 95% CI: 1.082–2.752, *P* = 0.022), heterozygous (OR: 1.430, 95% CI: 1.048–1.951, *P* = 0.024) and additive (OR: 1.348, 95% CI: 1.084–1.676, *P* = 0.007) genetic models. No significant association between *TAP1* I333V gene polymorphism and T1DM was detected in a recessive genetic model (OR: 1.384, 95% CI: 0.743–2.579, *P* = 0.306) in the entire population, especially among Caucasians. No significant association between them was found in an Asian or African population. *TAP1* I333V gene polymorphism was significantly associated with increased T1DM risk. V allele carriers might be predisposed to T1DM susceptibility.

## Introduction

Diabetes mellitus (DM) is a common dysmetabolic syndrome that causes internal insulin insufficiency or insulin resistance, and contributes to the glucose and lipid metabolism disturbance led by the combined action of genetic and environmental factors. Type 1 DM (T1DM), also known as insulin-dependent DM (IDDM), is an organ-specific autoimmune disease characterized by selective destruction of the insulin-producing islet beta cells of the pancreas [1]. Type 1 diabetes mellitus patients account for 7–10% of DM patients. The clinical symptoms of T1DM are relatively serious, and harm the physical and psychological health of patients. The pathogenesis of T1DM is highly complex. The β cell autoantigens, cells from the autoimmune system (such as T cells, B cells, macrophagocytes and dendritic cells), and cytokines and radicals produced by these cells have a critical function in the progression of T1DM.

Human leucocyte antigen (HLA) region, also known as major histocompatibility complex (MHC) region, located in 6p21, are the major gene regions for T1DM. Approximately 60% of T1DM susceptibility is attributed to HLA gene, in which class II region is most strongly associated with T1DM. A total of 42% of T1DM susceptibility comes from this region. *Transporter associated with antigen processing* (*TAP*) gene is located in the class II region between DQB1 and DPB1. The *TAP1* gene product is a transporter associated with the endogenous antigen processing and presentation. The TAP1 can transport peptides into the endoplasmic reticulum, where they combine with MHC I molecules. The MHC I molecules and antigen peptide compounds are subsequently expressed in the cytomembrane and recognized by the CD8^+^ T cell receptor, thereby stimulating a cytotoxic lymphocyte protective immune response [2]. In 1992, Van Kaer *et al*. found that MHC I molecules are significantly reduced and depleted of CD8^+^ T cells in transgenic mice with a disrupted *TAP1* gene [3]. *TAP1* gene deficiency and mutation might lead to the endogenous antigen processing and transportation barrier, which results in such autoimmune disease as T1DM. The 1207th base adenine (A) of the *TAP1* gene is substituted by guanine (G), which results in the wild-type isoleucine (Ile, I) being replaced by valine (Val, V) at 333rd amino acid.

Although some studies on the relationship between *TAP1* I333V gene polymorphism and T1DM exist, the results from individual studies remain controversial. In 1994, Cucca *et al*. reported that no primary association exists between *TAP1* I333V gene polymorphism and IDDM in Italy [4]. Analogously, Nakanishi *et al*. found that no significant difference existed in the distribution of *TAP1* I333V alleles between IDDM patients and normal controls, and they concluded that *TAP1* I333V gene polymorphism did not exhibit a primary association with Japanese IDDM [5]. In contrast, Jackson *et al*. determined the relative risk of *TAP1* I333V gene polymorphism and T1DM using single-stranded conformation polymorphism in the United States [6]. In 2004, Sheng *et al*. reported that *TAP1* 333V allele was the susceptibility allele for T1DM in a Chinese population [7].

In this study, a meta-analysis involving 1140 T1DM patients and 1108 controls was conducted to determine the relationship between *TAP1* I333V gene polymorphism and T1DM.

## Materials and methods

### Publication search and inclusion criteria

The terms ‘*Transporter associated with antigen processing 1*’, ‘I333V’, ‘T1DM’ and ‘polymorphism’ were adopted to search the electronic databases of PubMed, Embase, Web of Science, China National Knowledge Infrastructure and China Biological Medicine Database. The last research was updated on 31 October 2013, and the included publication years ranged from 1992 to 2007.

The chosen studies had to be in accordance with the following criteria: (*i*) evaluation of the *TAP1* I333V gene polymorphism and T1DM; (*ii*) T1DM diagnosis and classification criteria were presented by the American Diabetes Association and revised by the World Health Organization in 1997; (*iii*) case–control or cohort study published in an official journal; and (*iv*) the study should follow the Hardy–Weinberg equilibrium (HWE).

### Data extraction

Data were abstracted according to a standard protocol. Studies that did not meet the inclusion criteria, were repeated publications, or provided deficient data were eliminated. If similar data appeared in different studies, the data were only used once. The abstracted data contained the following items: the first author*s name, publication year, region, number of genotypes, genotyping, study design, matching criteria, and total number of cases and controls.

### Statistical analyses

Six genetic models, namely, the allelic (distribution of V allelic frequency of *TAP1* I333V gene polymorphism), recessive (VV *versus* IV+II), dominant (IV+VV *versus* II), homozygous (VV *versus* II), heterozygous (IV *versus* II) and additive (V *versus* I) genetic models, were adopted. The relationship between *TAP1* I333V gene polymorphism and T1DM was compared using odds ratio (OR) and its corresponding 95% confidence interval (CI). Chi-squared-based Q-test was used to calculate the heterogeneity between the individual studies, and significance was set at *P* < 0.05 [8]. If heterogeneity was observed among the individual studies, the pooled OR was estimated using a random-effect model (DerSimonian and Laird method) [9]. Otherwise, a fixed-effect model was used (Mantel-Haenszel method) [10]. The pooled OR was determined by Z-test, and significance was also set at *P* < 0.05.

Hardy–Weinberg equilibrium was assessed by Fisher*s exact test, and significance was set at *P* < 0.05. The funnel plot was used to estimate the potential publication bias. Egger*s linear regression test on the natural logarithm scale of the OR was used to assess funnel plot asymmetry, and significance was set at *P* < 0.05 [11]. STATA 11.0 software was used for statistical analyses (StataCorp, College Station, TX, USA).

## Results

### Studies and populations

A total of 25 studies were searched, among which 13 papers fulfilled the inclusion criteria. Data were abstracted from 1140 T1DM cases and 1106 controls (Table 1) [4–7,12–20]. The nine study regions were China, Italy, Denmark, France, Japan, United States, Germany, Senegal and Finland. These countries belong to four continents, namely, Asia, America, Europe and Africa. The populations from America and Europe belonged to the Caucasian subgroup. The remaining studies belonged to the Asian and African subgroups. Among the 12 excluded studies, five were reviews [21–25], and seven studies were not associated with *TAP1* I333V gene polymorphism or T1DM [26–32]. No study was discarded for deviating from HWE.

**Table 1:** Characteristics of the investigated studies of the association of the TAP1 I333V gene polymorphism and T1DM

				T1DM			Control					
								
Author	Year	Region	Ethnicity	II	IV	VV	II	IV	VV	Geno-typing	Matching criteria	Sample size (T1DM/control)
Colonna [12]	1992	Italy	Caucasian	29	17	4	27	10	0	PCR-SSCP	Ethnicity	50/37
Caillat-Zucman [13]	1993	France	Caucasian	74	35	3	66	31	1	PCR-SSOM	Ethnicity	112/98
Jackson [6]	1993	USA	Caucasian	147	75	19	143	54	4	PCR-SSCP	Ethnicity	241/201
Cucca [4]	1994	Italy	Caucasian	99	26	4	52	25	2	ARMS-PCR	Ethnicity	129/99
Kawaguchi [14]	1994	Japan	Asia	36	9	0	45	7	1	PCR-RFLP	Age, sex, ethnicity	45/53
Nakanishi [5]	1994	Japan	Asia	77	18	0	59	15	1	PCR-SSOM	Ethnicity	95/75
van Endert [15]	1994	Denmark	Caucasian	19	27	1	40	21	1	PCR-SSOM	Ethnicity	47/62
Chauffert [16]	1997	Senegal	Africa	55	34	3	79	36	2	PCR-RFLP	Sex, ethnicity	92/117
Ma [17]	1997	Finland	Caucasian	77	39	3	66	23	3	ARMS-PCR	Age, sex, ethnicity	119/92
Rau [18]	1997	German	Caucasian	40	54	4	82	68	11	PCR-SSCP	Ethnicity	98/161
Yan [19]	1997	USA	Caucasian	36	10	3	21	7	2	PCR-RFLP	Ethnicity	49/30
Sheng [7]	2004	China	Asia	12	35	0	36	13	4	PCR	Sex, ethnicity	47/53
Zhai [20]	2007	China	Asia	10	3	3	40	7	1	PCR-RFLP	Sex, ethnicity	16/48

T1DM: type 1 diabetes mellitus; PCR-RFLP: polymerase chain reaction-restriction fragment length polymorphism; ARMS-PCR: amplification refractory mutation system PCR; PCR-SSOM: PCR sequence specific oligonucleotide method; PCR-SSCP: PCR-single-strand conformation polymorphism; NP: not provided. Case–control study design was used in all of the above studies.

### Pooled analyses

**Fig. 1:**
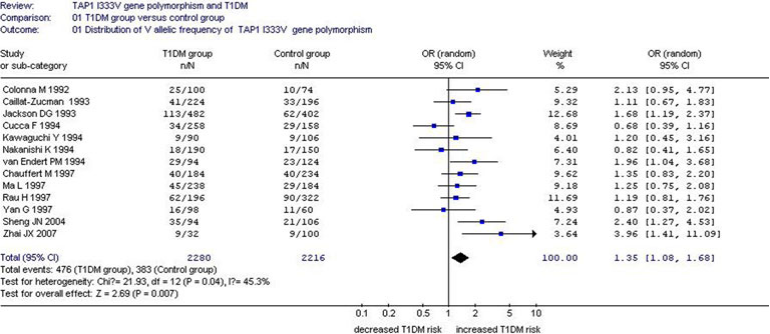
Forest plot of T1DM associated with *TAP1* I333V gene polymorphism under an allelic genetic model (distribution of V allelic frequency of *TAP1* gene).

**Fig. 2:**
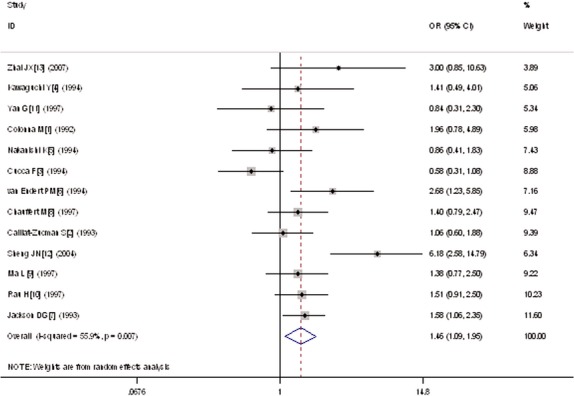
Forest plot of T1DM associated with *TAP1* I333V gene polymorphism under a dominant genetic model (VV+ IV *versus* II).

**Fig. 3:**
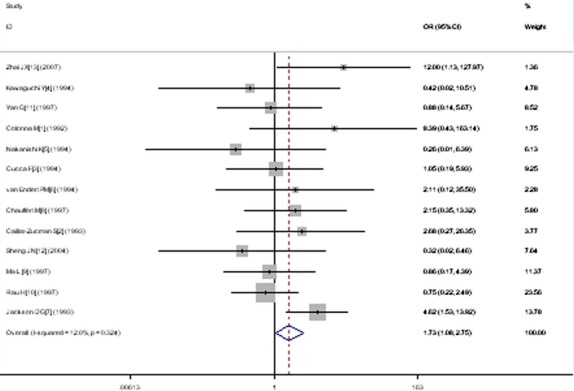
Forest plot of T1DM associated with *TAP1* I333V gene polymorphism under a homozygous genetic model (VV+ IV *versus* II).

**Fig. 4:**
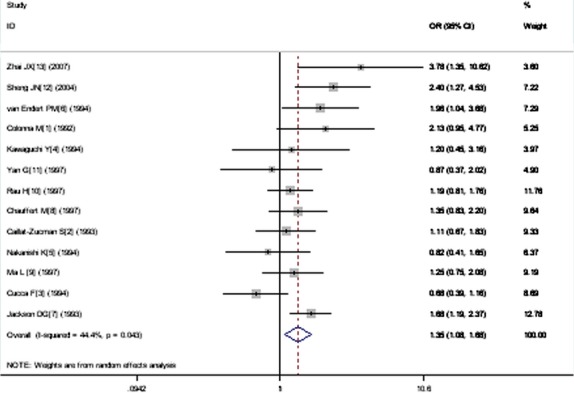
Forest plot of T1DM associated with *TAP1* I333V gene polymorphism under an additive genetic model (V *versus* I).

A significant relationship was observed between *TAP1* I333V gene polymorphism and T1DM in allelic (OR: 1.35, 95% CI: 1.08–1.68, *P* = 0.007), dominant (OR: 1.462, 95% CI: 1.094–1.955, *P* = 0.010), homozygous (OR: 1.725, 95% CI: 1.082–2.752, *P* = 0.022), heterozygous (OR: 1.430, 95% CI: 1.048–1.951, *P* = 0.024) and additive (OR: 1.348, 95% CI: 1.084–1.676, *P* = 0.007) genetic models. No significant association between *TAP1* I333V gene polymorphism and T1DM was detected in a recessive genetic model (OR: 1.384, 95% CI: 0.743–2.579, *P* = 0.306) in the entire population, especially among Caucasians. No significant association was found between them among the Asian or African population (Figs 1–4, Table 2).

**Table 2:** Summary of meta-analysis of association of *TAP1* I333V gene polymorphism and T1DM

Genetic model	Pooled OR (95% CI)	P value	Literature number	T1DM size	Control size	*P*_heterogeneity_ (*I*^2^%)
Allelic genetic model	1.35 (1.08–1.68)	0.007*	13	1140	1106	0.04* (44.4)
Subgroup 1: before 1995	1.26 (0.90–1.75)	0.17	7	719	605	0.04* (53.7)
Subgroup 2: after 1996	1.39 (1.11–1.74)	0.004*	6	421	501	0.13 (40.8)
Recessive genetic model	1.384 (0.743–2.579)	0.306	13	1140	1106	0.202 (23.9)
Dominant genetic model	1.462 (1.094–1.955)	0.010*	13	1140	1106	0.007* (55.9)
Subgroup 1: IV1 < 30	1.289 (0.781–2.126)	0.320	7	431	384	0.034* (56.1)
Subgroup 2: IV1 > 30	1.620 (1.143–2.296)	0.007*	6	709	722	0.041* (56.9)
Homo genetic model	1.725 (1.082–2.752)	0.022*	13	1140	1106	0.324 (12.0)
Hetero genetic model	1.430 (1.048–1.951)	0.024*	13	1140	1106	0.004* (58.4)
Subgroup 1:II1 < 40	2.183 (1.148–4.151)	0.017*	6	254	283	0.037* (57.8)
Subgroup 2: II1 ≥ 40	1.191 (0.963–1.472)	0.106	7	886	823	0.190 (31.2)
Additive genetic model	1.348 (1.084–1.676)	0.007*	13	1140	1106	0.043 (44.4)
Subgroup 1:II1 < 40	1.867 (1.361–2.561)	<0.001*	6	254	283	0.262 (22.8)
Subgroup 2: II1 ≥ 40	1.218 (1.023–1.450)	0.027*	7	886	823	0.147 (36.8)

* *P* < 0.05. T1DM: type 1 diabetes mellitus; CI: confidence interval; OR: odds ratio; T1DM size: the total number of T1DM cases; control size: the total number of control group; homo genetic model: homozygous genetic model; hetero genetic model: heterozygous genetic model.

Significant heterogeneity was observed in the allelic (P_heterogeneity_ = 0.04), dominant (P_heterogeneity_ = 0.007), heterozygous (P_heterogeneity_ = 0.004) and additive (P_heterogeneity_ = 0.043) genetic models. The following meta-regression was performed to explore the source of heterogeneity. In the allelic genetic model, the heterogeneity could only be explained by the publication year (*P* = 0.026). The entire population was separated into two subgroups based on publication year. The manuscripts published before 1995 belonged to subgroup 1, and the manuscripts published after 1996 were classified into subgroup 2. In subgroup analysis stratified by publication year, significantly increased T1DM risk was detected in subgroup 2 (OR: 1.39, 95% CI: 1.11–1.74, *P* = 0.004), whereas no significant increase in T1DM risk was found in subgroup 1 (OR: 1.26, 95% CI: 0.90–1.75, *P* = 0.17). Heterogeneity was observed in subgroup 1 (P_heterogeneity_ = 0.04, *I*^2^ = 53.7%), but not in subgroup 2 (P_heterogeneity_ = 0.13, *I*^2^ = 40.8%). These results indicate that heterogeneity mainly existed in the studies published before 1995, and the conclusions were relatively consistent in the studies published after 1996.

In the dominant genetic model, heterogeneity could be explained by publication year (*P* = 0.039), II genotype number of T1DM group sample size (II1, *P* = 0.003), IV genotype number of T1DM group sample size (IV1, *P* = 0.001) and IV genotype number of control group sample size (IV0, *P* = 0.023). All the individual studies were classified into two subgroups according to IV1. The studies with IV1 < 30 were grouped into subgroup 1, whereas the studies with IV1 > 30 were grouped into subgroup 2. In the subsequent subgroup analysis stratified by IV1, significantly increased T1DM risk was only found in subgroup 2 (OR: 1.620, 95% CI: 1.143–2.296, *P* = 0.007). In subgroup 1, no significant increase in T1DM risk was detected (OR: 1.289, 95% CI: 0.781–2.126, *P* = 0.320). Heterogeneity decreased in two subgroups but remained significant (subgroup 1: P_heterogeneity_ = 0.034, *I*^2^ = 56.1%; subgroup 2: P_heterogeneity_ = 0.041, *I*^2^ = 56.9%).

In the heterozygous genetic model, heterogeneity could be explained by II1 (*P* = 1.0 × 10^−10^), IV0 (*P* = 0.005), total sample size of control group (T0, *P* = 0.028) and weight (*P* = 0.002). All the individual studies were divided into two subgroups according to II1. Subgroup 1 was defined as II1 < 40, and subgroup 2 was defined as II1 ≥ 40. In the subsequent subgroup analysis stratified by II1, significantly increased T1DM risk was detected in subgroup 1 (OR: 2.183, 95% CI: 1.148–4.151, *P* = 0.017, P_heterogeneity_ = 0.037). In subgroup 2, no significant increase in T1DM risk was found (OR: 1.191, 95% CI: 0.963–1.472, *P* = 0.106, P_heterogeneity_ = 0.190).

In the additive genetic model, heterogeneity could be explained by II1 (*P* = 0.003), VV genotype number of T1DM group sample size (VV1, *P* = 0.027) and VV genotype number of control group sample size (VV0, *P* = 0.027). Individual studies were separated into two subgroups according to II1, which was similar to that in the heterozygous genetic model. In the following subgroup analysis stratified by II1, significantly increased T1DM risk was detected in both subgroups (subgroup 1: OR: 1.867, 95% CI: 1.361–2.561, *P* < 0.001; subgroup 2: OR: 1.218, 95% CI: 1.023–1.450, *P* = 0.027). The heterogeneity disappeared in both subgroups (subgroup 1: P_heterogeneity_ = 0.262, *I*^2^ = 22.8%; subgroup 2: P_heterogeneity_ = 0.147, *I*^2^ = 36.8%).

Logistic regression was performed on multivariable-adjusted risks, such as age, sex, region and ethnicity. These risk factors had no effect on the association between *TAP1* I333V gene polymorphism and T1DM (OR = 1).

In the subgroup analysis stratified by ethnicities among the Caucasian population, a significant association between *TAP1* I333V gene polymorphism and T1DM was found in allelic (OR: 1.30, 95% CI: 1.09–1.55, *P* = 0.004), dominant (OR: 1.330, 95% CI: 1.079–1.640, *P* = 0.008), homozygous (OR: 1.854, 95% CI: 1.082–3.176, *P* = 0.022), heterozygous (OR: 1.351, 95% CI: 0.889–2.054, *P* = 0.029) and additive genetic models (OR: 1.297, 95% CI: 1.088–1.548, *P* = 0.004). No significant association between *TAP1* I333V gene polymorphism and T1DM was detected in a recessive genetic model (OR: 1.643, 95% CI: 0.971–2.780, *P* = 0.064).

In the Asian subgroup, no significant association between *TAP1* I333V gene polymorphism and T1DM was found in the allelic (OR: 1.69, 95% CI: 0.86–3.34, *P* = 0.13), recessive (OR: 0.928, 95% CI: 0.222–3.873, *P* = 0.918), dominant (OR: 2.133, 95% CI: 0.815–5.583, *P* = 0.123), homozygous (OR: 1.235, 95% CI: 0.293–5.211, *P* = 0.773) and heterozygous genetic models (OR: 2.141, 95% CI: 0.773–6.248, *P* = 0.164). A significant association was found between them in a additive genetic model (OR: 1.614, 95% CI: 1.095–2.378, *P* = 0.015; Table 3).

**Table 3:** Summary of meta-analysis of association of *TAP1* I333V gene polymorphism and T1DM stratified by ethnicities

Genetic model	Pooled OR (95% CI)	P value	Study number	T1DM size	Control size	*P*_heterogeneity_ (*I*^2^%)
Allelic genetic model	1.35 (1.08–1.68)	0.007*	13	1140	1106	0.04* (44.4)
Caucasian subgroup	1.30 (1.09–1.55)	0.004*	8	845	760	0.09 (43.3)
Asian subgroup	1.69 (0.86–3.34)	0.13	4	203	229	0.04* (64.2)
African subgroup	1.35 (0.83–2.20)	0.23	1	92	117	NA
Recessive genetic model	1.384 (0.743–2.579)	0.306	13	1140	1106	0.202 (23.9)
Caucasian subgroup	1.643 (0.971–2.780)	0.064	8	845	760	0.301 (16.4)
Asian subgroup	0.928 (0.222–3.873)	0.918	4	203	229	0.071 (57.3)
African subgroup	0.900 (0.578–1.399)	0.639	1	92	117	NA
Dominant genetic model	1.462 (1.094–1.955)	0.010*	13	1140	1106	0.007* (55.9)
Caucasian subgroup	1.330 (1.079–1.640)	0.008*	8	845	760	0.074 (45.8)
Asian subgroup	2.133 (0.815–5.583)	0.123	4	203	229	0.007 (75.0)
African subgroup	1.399 (0.792–2.470)	0.248	1	92	117	NA
Homozygous genetic model	1.725 (1.082–2.752)	0.022*	13	1140	1106	0.324 (12.0)
Caucasian subgroup	1.854 (1.082–3.176)	0.025*	8	845	760	0.349 (10.5)
Asian subgroup	1.235 (0.293–5.211)	0.773	4	203	229	0.129 (47.1)
African subgroup	3.136 (0.509–19.315)	0.218	1	92	117	NA
Heterzygous genetic model	1.430 (1.048–1.951)	0.024*	13	1140	1106	0.004* (58.4)
Caucasian subgroup	1.351 (0.889–2.054)	0.029*	8	845	760	0.081 (44.7)
Asian subgroup	2.141 (0.733–6.248)	0.164	4	203	229	0.004* (77.2)
African subgroup	1.975 (1.119–3.486)	0.019^*^	1	92	117	NA
Additive genetic model	1.348 (1.084–1.676)	0.007*	13	1140	1106	0.043* (44.4)
Caucasian subgroup	1.297 (1.088–1.548)	0.004*	8	845	760	0.09 (43.3)
Asian subgroup	1.614 (1.095–2.378)	0.015*	4	203	229	0.044* (62.9)
African subgroup	1.347 (0.827–2.196)	0.232	1	92	117	NA

NA: not applicable.

* *P* < 0.05.

**Fig. 5:**
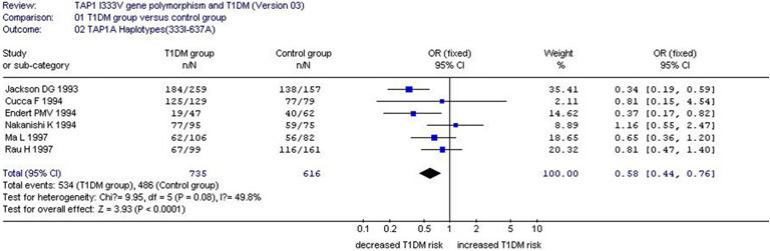
Forest plot of T1DM associated with *TAP1A haplotype* (333I-637A).

The haplotypes from *TAP1* I333V and Asp637Gly (A637G) gene polymorphisms were chosen, constructed and analysed. The three haplotypes, namely, *TAP1A* (333I-637A), *TAP1B* (333V-637G) and *TAP1C* (333V-637A) haplotypes, were constructed and analysed. In the following meta-analysis, the *TAP1A* haplotype could decrease T1DM risk (OR: 0.58, 95% CI: 0.44–0.76, *P* < 0.001). The other two haplotypes, namely, *TAP1B* and *TAP1C*, had no association with T1DM risk (*TAP1B*: OR: 1.12, 95% CI: 0.56–2.23, *P* *=* 0.75; *TAP1C*: OR: 1.79, 95% CI: 0.43–7.53, *P* = 0.43; Fig. 5, Table 4).

**Table 4:** Summary of meta-analysis of association of TAP1 I333V and Asp637Gly (A637G) haplotypes

Genetic model	Pooled OR (95% CI)	P value	Study number	T1DM size	Control size	*P*_heterogeneity_ (*I*^2^%)
TAP1A (333I-637A)	0.58 (0.44–0.76)	<0.001*	6	735	616	0.08 (49.8)
TAP1B (333V-637G)	1.12 (0.56–2.23)	0.75	5	847	685	0.009 (70.5)
TAP1C (333V-637A)	1.79 (0.43–7.53)	0.43	3	635	507	0.007* (80.0)

* *P* < 0.05.

### Bias diagnostics

**Fig. 6:**
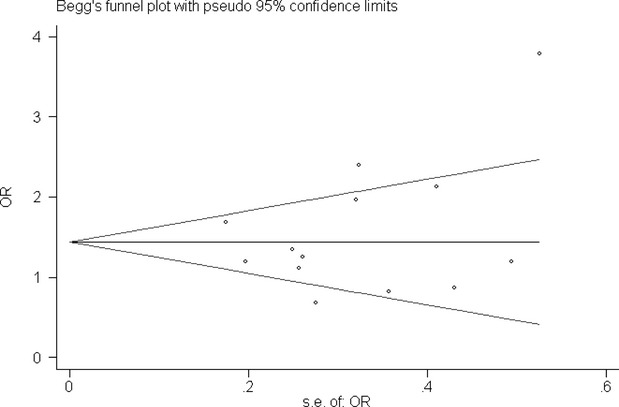
Begg*s funnel plot for studies of the association of T1DM associated with *TAP1* I333V gene polymorphism under an additive genetic model (V *versus* I). The horizontal and vertical axis correspond to the OR and confidence limits. OR: odds ratio; SE: standard error.

The funnel plot and Egger*s test were used to evaluate the publication bias of the individual studies. No publication bias was observed by visual inspection of the Begg*s funnel plot in the additive genetic model (Fig. 6). No significant difference in Egger*s test was observed, which implies low publication bias in this meta-analysis (additive genetic model, T = 0.90, *P* = 0.386).

## Discussion

In this meta-analysis, a significant relationship was observed between *TAP1* I333V gene polymorphism and T1DM in the allelic (OR: 1.35), dominant (OR: 1.462), homozygous (OR: 1.725), heterozygous (OR: 1.430) and additive (OR: 1.348) genetic models, especially among Caucasians. No positive association between *TAP1* I333V gene polymorphism and T1DM was observed in a recessive genetic model (OR: 1.384), which could be associated with the less VV genotype number in the general population. Table 1 showed that no VV genotype was present in several studies. Thus, *TAP1* 333V allele possibly increased T1DM risk, i.e. V allele possibly conferred T1DM susceptibility to humans. No significant association was found between *TAP1* I333V gene polymorphism and T1DM in an Asian or African population. The different results from the different populations were probably associated with the different ethnicities. Moreover, the relatively few papers on Asian and African populations possibly led to the negative results, which implied that large-scale studies should be performed in future.

Given that heterogeneity existed in the allelic and heterozygote genetic models, meta-regression was performed to determine the source of heterogeneity. In the subsequent heterogeneity source analysis, publication year, IV1 and II1 were suggested to be the central confounding factors to explain the source of heterogeneity in allelic, dominant, heterozygous and additive genetic models (*P* < 0.05).

*Transporter associated with antigen processing 1* A637G is another common *TAP1* gene polymorphism associated with T1DM. In the *TAP1* gene, 2120th base adenine (A) was replaced by guanine (G), which resulted in the corresponding amino acid aspartate (Asp, A) being substituted by glycine (Gly, G) at 637th amino acid, of which 637G allele possibly increased the T1DM risk. Thus, analysis of the *TAP1* gene 333IV-637AG haplotypes was performed [6,7]. The results from haplotype analysis showed that *TAP1A* (333I-637A) haplotype had a protective effect for T1DM, which was in agreement with the aforementioned meta-analysis results. The negative association between the *TAP1B* (333V-637G) and *TAP1C* (333V-637A) haplotypes and T1DM was possibly associated with the limited number of studies because only five and three papers were included respectively. Thus, the results need to be further verified by more studies.

*Transporter associated with antigen processing 1* gene polymorphisms result in different recognition and transport affinity to the same endogenous antigen peptide, and cause different bodies to show various immune responses to the same endogenous antigen, including protective immunity, immune tolerance and autoimmune tendency [33]. The mechanism of the association between *TAP1* I333V gene polymorphism and T1DM remains unclear. In 1998, Quadri *et al*. reported that *TAP1* 333I allele specifically enhances translocation of model peptides containing basic C-terminal amino acid residue. However, *TAP1* 333V allele did not show specificity for the peptide with basic amino acid residue C-terminus, which indicated that the *TAP1* I333V mutation could lead to the change in specificity of the transported peptides. Some peptides transported by mutated *TAP1* gene products might cause the expression deficiency of MHC I molecules, which resulted in the spontaneous termination of immune tolerance, launched the autoimmunity process and caused the destruction of pancreas islet β cells [34]. In the present meta-analysis, *TAP1* 333V allele increased the T1DM risk, which could be associated with the differences in peptide transport. However, this result is still a hypothesis that needs confirmation by future experiments.

In 2004, Sheng *et al*. found that the IV genotype of *TAP1* gene locus 333 was a susceptible gene for T1DM that had a possible function in the production of autoantibodies, such as insulin autoantibody, islet cell antibody and glutamic acid decarboxylase antibody. Thus, the autoimmune response and destruction in the pancreas islet β cells resulted in T1DM. However, the concrete mechanisms by which the *TAP1* 333 IV genotype promoted autoantibody production remained unclear [7].

The following limitations were noted in the present meta-analysis. Large-scale studies on the association of T1DM with *TAP1* I333V gene polymorphism are still insufficient. The TAP1 expression level was influenced not only by *TAP1* I333V gene polymorphism but also by other genetic and environmental factors, such as ethnicity, inflammation state and other immune system diseases. Given that T1DM is a multigenic heredity disease, *TAP1* I333V gene polymorphism might be associated with the gene linkage disequilibrium, such as *TAP1* Val458Leu, Asp637Gly and Arg648Gln, which increase T1DM susceptibility [35].

*Transporter associated with antigen processing 1* I333V gene polymorphism was positively associated with increased T1DM risk. Patients with the V allele may be susceptible to T1DM. Our results may help in establishing individual T1DM therapy strategies. In consideration of the limitations, more large-scale studies are needed to elucidate the significance of our conclusions.
